# Antenatal depressive symptoms and adverse perinatal outcomes

**DOI:** 10.1186/s12884-021-03783-9

**Published:** 2021-04-20

**Authors:** Despina Pampaka, Stefania I. Papatheodorou, Mohammad AlSeaidan, Rihab Al Wotayan, Rosalind J. Wright, Julie E. Buring, Douglas W. Dockery, Costas A. Christophi

**Affiliations:** 1 Cyprus International Institute for Environmental and Public Health, Cyprus University of Technology, 95 Eirinis Street, 3041 Limassol, Cyprus; 2 Department of Epidemiology, Harvard T.H. Chan School of Public Health, Boston, MA USA; 3 Dasman Diabetes Institute, Kuwait, Kuwait; 4 Primary Health Care, Ministry of Health, Kuwait, Kuwait; 5 Department of Pediatrics & Institute for Exposomic Research, Icahn School of Medicine at Mount Sinai, New York, NY USA; 6 Division of Preventive Medicine, Department of Medicine, Brigham and Women’s Hospital and Harvard Medical School, Boston, MA USA; 7 Department of Environmental Health, Harvard T.H. Chan School of Public Health, Boston, MA USA

**Keywords:** Antenatal depressive symptoms, Preterm birth, Small for gestational age, Large for gestational age, Adverse perinatal outcomes, Kuwait

## Abstract

**Background:**

The association of antenatal depression with adverse pregnancy, birth, and postnatal outcomes has been an item of scientific interest over the last decades. However, the evidence that exists is controversial or limited. We previously found that one in five women in Kuwait experience antenatal depressive symptoms. Therefore, the aim of this study was to examine whether antenatal depressive symptoms are associated with preterm birth (PTB), small for gestational age (SGA), or large for gestational age (LGA) babies in this population.

**Methods:**

This was a secondary analysis based on data collected in the Transgenerational Assessment of Children’s Environmental Risk (TRACER) Study that was conducted in Kuwait. Logistic regression analysis was used to examine whether antenatal depressive symptoms assessed using the Edinburgh Depression Scale (EDS) were associated with preterm birth, small for gestational age, and large for gestational age babies.

**Results:**

A total of 1694 women had complete information about the outcomes of interest. Women with depressive symptoms in pregnancy had increased, albeit non-significant, odds of having PTB (OR = 1.41; 95%CI: 0.81, 2.45), SGA babies (OR = 1.26; 0.80, 1.98), or LGA babies (OR = 1.27; 0.90, 1.79). Antenatal depressive symptoms had similar increased odds for the three outcomes even after adjusting for several covariates though none of these reached statistical significance.

**Conclusions:**

In the present study, the depressive symptoms in pregnancy did not predict adverse birth outcomes, such as PTB, SGA, and LGA, which adds to the currently non-conclusive literature. However, further research is needed to examine these associations, as the available evidence is quite limited.

## Introduction

Every year approximately 15 million babies are born preterm [1]. Several complications are associated with preterm delivery making it the leading cause of perinatal mortality and a major cause of child death in many middle and high income countries [2]. Preterm birth (PTB) puts a serious burden on the healthcare system as babies who are born prematurely present both short and long term complications and are at a higher risk of morbidity [3]. Other adverse perinatal outcomes such as low birth weight (LBW), small for gestational age (SGA), and large for gestational age (LGA) have also a direct or indirect impact on the health of the newborn and may result in long-term sequelae [4–7].

These adverse events are complex and are associated with a number of factors, including biological, obstetrical, behavioral, psychological, and socio-economic. Over the past two decades, there has been an increasing interest in the association of antenatal depression with adverse perinatal outcomes [8–10]. Several studies examined the effect of depression in pregnancy on preterm birth (PTB) and found a positive association [11–14]. Furthermore, antenatal depression has been identified as a risk factor for low birth weight (LBW) [13]. These associations have also been presented in the meta-analyses of Grote et al. who computed a pooled RR of 1.39 (95% CI: 1.19, 1.61) and 1.49 (95% CI: 1.25, 1.77) for the association of PTB and LBW, respectively [8]. In addition, women with depression in pregnancy, especially in mid-pregnancy, have been shown to experience greater odds of delivering a baby with small for gestational age (SGA) [9, 15]. At the same time, the evidence about the association of antenatal depression and macrosomia (> 4000 g) or large for gestational age babies (LGA) is very limited [9]. Despite the fact that there is some scientific evidence which supports that antenatal depression is associated with these adverse outcomes, these associations are not consistent, even when comparing results from studies conducted in the same country [10, 16–19].

The mechanism underlying the association of depression and psychosocial stress with birth weight is also not clear [20]. Cortisol levels in pregnancy are shown to be inversely proportional to birth weight but these levels cannot be explained by prenatal stress [21]. Different mechanisms have been suggested to explain the possible association of depression and PTB. Depressed mood is linked to a decrease in the activity of natural killer cells and a rise in the plasma levels of inflammatory cytokines, suggesting that the effect of depression on PTB is mediated through inflammation [22]. Moreover, the depression-PTB association could be mediated by behavioral factors related to depression. Depressive symptoms in pregnancy have been associated with adverse health habits, such as smoking and alcohol consumption, which are known risk factors for PTB [2, 23].

Other psychosocial problems that may exist before or during pregnancy have been also described as potential risk factors of perinatal adverse outcomes. A study conducted among a group of African-American women in the USA suggested that a poor psychosocial profile was significantly associated with preterm delivery as well as low birth weight infants [20]. Women with post-traumatic stress disorder (PTSD) were also shown to be at a greater risk of preterm birth [24]. Shapiro et al. summarized the results from different studies and concluded that psychosocial stress is associated with preterm birth, with perceived stress and pregnancy-related anxiety being the two indicators most consistently reported to increase the risk of PTB [25].

AlSeaidan et al. (2016) looked at birth outcomes in a prospective pregnancy-birth cohort study in Kuwait and found that the prevalence of PTB and SGA was similar to other developed countries, while macrosomia and LGA were in fact greater than what it was expected [26]. The authors also reported that SGA and LGA were associated with pre-pregnancy maternal overweight or obesity. In a secondary analysis of data collected in this birth cohort, we estimated that as high as one in five women in Kuwait experiences depressive symptoms during pregnancy and these symptoms are usually comorbid with other indicators of a poor psychosocial profile [27].

Examining whether antenatal depression is associated with poor perinatal outcomes is important, as it will add important information in this limited or contradictory literature. Thus, the aim of this study is to investigate whether antenatal depressive symptoms predict preterm birth, small for gestational age or large for gestational age babies using data collected from a prospective cohort study in a population where antenatal depressive symptoms were found to be relatively common [26, 27].

## Methods

### Study design

This analysis is based on data from the Transgenerational Risk Assessment of Children’s Risk (TRACER) study that was conducted in Kuwait. Details of this study have been published elsewhere [26]. Briefly, the TRACER study is a longitudinal prospective birth cohort study that was set up by the Harvard T.H. Chan School of Public Health in the USA and the Dasman Diabetes Institute in Kuwait. The main aim of this study was to examine prenatal risk factors for early childhood obesity. The review boards of both institutions provided ethical approval for the study and permission for recruitment of participants was obtained from the participating health centres.

### Participants

A convenience sampling method was used, approaching pregnant women attending antenatal visits at primary public health clinics in each of the six governorates of Kuwait and three private clinics. The clinic staff provided a brochure and referred interested women to the onsite research assistant. The TRACER study was open to both Kuwaiti and non-Kuwaiti women attending public and private clinics, thus making the sample representative of the population in Kuwait. Women were eligible to participate if they were between 18 and 45 years old, had a singleton pregnancy, and were fluent in Arabic or English. Most of the women were enrolled in the second trimester of their pregnancy but they were also eligible to participate if they were in their first or third trimester. A written consent from the woman and her partner was required for participation in this study.

### Data collection

Data were collected from May 2012 until August 2015. The collection process included several interviewer-administered questionnaires. For the purpose of the main research question of this analysis we used data collected using the Baseline questionnaire, which assessed the socio-demographic background and the medical history of women, including their last menstrual period; and the Stress questionnaire, which assessed several mental and physical health indicators before and during pregnancy, including antenatal depressive symptoms (Edinburgh Depression Scale). The Baseline questionnaire was administered at enrolment during a prenatal visit to the clinic or hospital while the Stress questionnaire was administered at a visit subsequent to enrolment or by a phone interview. In the event that the woman was recruited during the third trimester of her pregnancy, the Baseline and Stress questionnaires were both administered at enrolment. The enrolled participants were also contacted via phone at a median time of 6 weeks (IQR: 3–9) after delivery to obtain information about the birth date and birth weight and, at the same contact, the majority of the women also answered a postnatal questionnaire which included questions about the health and the diet of the baby, including breastfeeding. During the same call, the mother was asked to report any diagnosis and treatment for gestational diabetes and gestational hypertension in her last pregnancy.

### Measures

#### Preterm birth

The date of the last menstrual period and the birth date were used to calculate the gestational age at birth. PTB was defined as a gestational age at birth that was less than 37 weeks [28].

#### Small and large for gestational age babies

SGA was estimated based on the World Health Organization birth weight percentiles for gestational week [29]. If the birth weight was lower than the 10th percentile for the gestational age then the neonate was characterized as SGA. Similarly, LGA was defined by a birth weight greater than the 90th percentile [30].

#### Antenatal depressive symptoms

The main exposure of interest was the experience of antenatal depressive symptoms which were assessed during pregnancy using the Edinburgh Depression Scale (EDS). This is the same tool as the Edinburgh Postnatal Depression Scale (EPDS) which was originally developed to assess postnatal depression. However, some studies showed that subscales of the EPDS could also screen for anxiety and anhedonia [31]. The tool has 10 items, which take a score of 0–3 each with 3 corresponding to higher depression or anxiety. Different cut-off points for defining the presence of depressive symptoms have been recommended in the literature. The questionnaire was not validated in this study population, therefore the cut-off point of EDS ≥ 10 was used to define depressive symptoms, similar to other multi-ethnic studies [32, 33].

#### Other variables

Apart from the aforementioned variables, we also included socio-demographic characteristics, such as age, nationality (Kuwaiti vs. non-Kuwaiti), employment status, educational level, and monthly household income in Kuwaiti Dinars [1 Kuwaiti Dinar (KWD) ≈ 3.5 United States Dollars (USD)]. We further examined the role of pre-pregnancy BMI and self-reported factors describing maternal reproductive health, such as parity, preterm delivery in a previous pregnancy, conception by in-vitro fertilization (IVF), as well as gestational diabetes and gestational hypertension in the current pregnancy.

### Statistical analysis

The strength of the linear association between gestational length and birth weight with the EDS scores was described using Spearman correlation coefficients. We also examined the association of depressive symptoms in pregnancy with each of the three outcomes of interest (PTB, SGA and LGA) using the chi-square test of independence and univariate logistic regression models and reported crude odds ratios (ORs) and the corresponding 95% confidence intervals (95% CI). The same analysis was repeated for socio-demographic characteristics, behavioural, reproductive health, obstetric, and psychosocial variables in order to identify potential risk factors for each of the outcomes of interest. We considered variables based on previous knowledge and biological plausibility. This was followed by a multivariable logistic regression model for each outcome to examine the adjusted effects of the variables considered. Each model included antenatal depressive symptoms, as this was the primary exposure of interest, along with variables that had a univariate association with a *p*-value< 0.25. Nationality and pre-pregnancy BMI were considered as potential effect-modifiers of the antenatal depressive symptoms associations with the outcomes. We checked for statistical significance of the possible effect-modifiers by adding interaction terms in the multivariable logistic regression models. The fit of the model was assessed using the Hosmer-Lemeshow goodness of fit test. All statistical analyses were conducted using SAS 9.3 (SAS Institute, Cary, NC, USA) and statistical significance was defined as a *p*-value< 0.05 using two-sided tests.

## Results

A total of 2038 women were enrolled in the TRACER study and completed the Baseline and Stress questionnaires with usable data. We excluded women without a score for the antenatal depressive symptoms (*n* = 92) and women who reported taking anxiety or depression medication during the current pregnancy (*n* = 6) or who completed the stress questionnaire in the first trimester (*n* = 24). We further restricted the sample to women who had provided information about their babies (*n* = 1798). Of these, a total of *n* = 1694 women had available data to calculate all PTB, SGA, and LGA and were included in the current analysis.

Among the 1129 multiparous women there were 469 (41.5%) with parity 2; 518 (45.9%) with parity 3–4; and 142 (12.6%) with parity > 4. The majority of the women in our sample were non-Kuwaitis (75.1%) and were younger than 30 years old (63.3%), similar in the group of women with depressive symptoms and the group without (Table 1). The two groups also had similar income, in vitro fertilization, as well as gestational hypertension and gestational diabetes rates. More than half (54.2%) had pre-pregnancy BMI in the overweight/obese group. The mean gestational age at delivery was 39.2 ± 1.7 weeks and the mean birth weight was 3216 ± 495 g. The group of women with depressive symptoms had somewhat smaller percentage unemployed than the group with no depressive symptoms, had lower education level and were more multiparous (all *p* < 0.05). Overall, the prevalence of PTB, SGA, and LGA in our sample was 7.3, 7.1, and 22.6%, respectively (Table 1) while the prevalence of depressive symptoms was 19.5%.

**Table 1:** Baseline and pregnancy-related characteristics

Characteristic	Overall (***N*** = 1694)	No depressive Symptoms (***N*** = 1363)	Depressive Symptoms (***N*** = 331)	***p***-value
***BASELINE***				
**Age group**				0.17
< 25	411 (24.3)	346 (25.4)	65 (19.6)	
25–30	660 (39.0)	520 (38.1)	140 (42.3)	
30–35	417 (24.6)	332 (24.4)	85 (25.7)	
> 35	206 (12.1)	165 (12.1)	41 (12.4)	
**Nationality**				0.22
Kuwaiti	421 (24.9)	330 (24.2)	91 (27.5)	
Non-Kuwaiti	1273 (75.1)	1033 (75.8)	240 (72.5)	
**Employment status**				0.04
Employed	765 (45.2)	622 (45.6)	143 (43.2)	
Housewife	759 (44.8)	594 (43.6)	165 (49.8)	
Unemployed	170 (10.0)	147 (10.8)	23 (7.0)	
**Education**				<.01
Up to high school	517 (30.5)	387 (28.4)	130 (39.3)	
Higher education	1177 (69.5)	976 (71.6)	201 (60.7)	
**Household Income (KWD)**^a^				0.17
< 400	499 (30.3)	388 (29.3)	111 (34.3)	
400–800	580 (35.2)	474 (35.8)	106 (32.7)	
800–1600	337 (20.4)	267 (20.2)	70 (21.6)	
≥ 1600	232 (14.1)	195 (14.7)	37 (11.4)	
***PREGNANCY***				
**Parity**				0.01
Primiparous	564 (33.3)	473 (34.7)	91 (27.5)	
Multiparous	1129 (66.7)	889 (65.3)	240 (72.5)	
**Pre-pregnancy BMI (kg/m**^**2**^**)**				0.85
< 18.5	39 (2.3)	31 (2.3)	8 (2.4)	
18.5–25	735 (43.6)	594 (43.7)	141 (43.0)	
25–30	551 (32.7)	448 (33.0)	103 (31.4)	
≥ 30	362 (21.4)	286 (21.0)	76 (23.2)	
**In vitro fertilization**				0.11
No	1652 (97.6)	1333 (97.9)	319 (96.4)	
Yes	41 (2.4)	29 (2.1)	12 (3.6)	
**Preterm delivery in previous pregnancies**				<0.01
No previous pregnancy	473 (27.9)	400 (29.3)	73 (22.0)	
No preterm delivery	112 (6.6)	80 (5.9)	32 (9.7)	
At least one preterm delivery	1108 (65.5)	882 (64.8)	226 (68.3)	
**Gestational hypertension**				0.58
No	1252 (95.3)	1015 (95.1)	237 (96.0)	
Yes	62 (4.7)	52 (4.9)	10 (4.0)	
**Gestational diabetes**				0.39
No	1228 (91.3)	988 (91.0)	240 (92.7)	
Yes	117 (8.7)	98 (9.0)	19 (7.3)	
**Sex of the baby**				0.78
Female	802 (47.3)	643 (47.2)	159 (48.0)	
Male	892 (52.7)	720 (52.8)	172 (52.0)	
**PTB**				0.38
No	1570 (92.7)	1267 (93.0)	303 (91.5)	
Yes	124 (7.3)	96 (7.0)	28 (8.5)	
**SGA**				0.13
No	1573 (92.9)	1272 (93.3)	301 (90.9)	
Yes	121 (7.1)	91 (6.7)	30 (9.1)	
**LGA**				0.43
No	1312 (77.4)	1061 (77.8)	251 (75.8)	
Yes	382 (22.6)	302 (22.2)	80 (24.2)	

^a^1 KWD ≈ 3.5 USD

**Table 2:** Baseline and pregnancy-related characteristics and outcomes of interest - Unadjusted ORs

Characteristic	PTB		SGA		LGA	
	OR	95% CI	OR	95% CI	OR	95% CI
***BASELINE***						
**Age group**						
< 25	0.98	0.61, 1.57	1.25	0.80, 1.97	0.67	0.49, 0.92
25–30	1.00	–	1.00	–	1.00	–
30–35	0.97	0.60, 1.55	0.76	0.46, 1.27	1.11	0.83, 1.48
> 35	0.98	0.54, 1.79	1.02	0.56, 1.87	1.55	1.09, 2.19
**Nationality**						
Kuwaiti	1.00	–	1.00	–	1.00	–
Non- Kuwaiti	0.58	0.39, 0.85	1.11	0.71, 1.71	1.38	1.05, 1.82
**Employment status**						
Employed	1.00	–	1.00	–	1.00	–
Housewife	0.90	0.61, 1.32	0.99	0.67, 1.46	0.97	0.76, 1.23
Unemployed	0.74	0.37, 1.47	0.79	0.40, 1.59	0.47	0.29, 0.76
**Education**						
Up to High School	0.93	0.62, 1.39	1.49	1.02, 2.18	0.94	0.74, 1.21
Higher education	1.00	–	1.00	–	1.00	–
**Household Income (KWD)**						
< 400	0.64	0.37, 1.12	2.04	1.09, 3.83	0.89	0.60, 1.32
400–800	0.60	0.35, 1.04	1.05	0.54, 2.03	1.35	0.93, 1.95
800–1600	0.86	0.48, 1.52	0.95	0.46, 1.98	1.41	0.95, 2.11
≥ 1600	1.00	–	1.00	–	1.00	–
***PREGNANCY***						
**Parity**						
Primiparous	0.92	0.62, 1.37	1.20	0.82, 1.76	0.54	0.42, 0.71
Multiparous	1.00	–	1.00	–	1.00	–
**Pre-pregnancy BMI**						
< 18.5	1.75	0.60, 5.15	1.26	0.43, 3.67	0.23	0.06, 0.97
18.5–25	1.00	–	1.00	–	1.00	–
25–30	1.46	0.96, 2.23	0.75	0.49, 1.15	1.46	1.12, 1.91
≥ 30	1.24	0.75, 2.03	0.61	0.36, 1.04	1.59	1.18, 2.14
**In vitro fertilization**						
No	1.00	–	1.00	–	1.00	–
Yes	4.39	2.10, 9.19	1.03	0.31, 3.38	1.27	0.63, 2.55
**Preterm delivery in previous pregnancies**						
No previous pregnancy	1.00	–	1.00	–	1.00	
No preterm delivery	0.99	0.64, 1.53	0.82	0.55, 1.23	1.80	1.35, 2.38
At least one preterm delivery	3.10	1.69, 5.68	1.00	0.47, 2.13	1.69	1.02, 2.78
**Gestational hypertension**						
No	1.00	–	1.00	–	1.00	–
Yes	1.84	0.81, 4.17	1.57	0.66, 3.75	1.65	0.96, 2.86
**Gestational diabetes**						
No	1.00	–	1.00	–	1.00	–
Yes	1.87	1.00, 3.48	1.08	0.51, 2.30	1.68	1.12, 2.54
**Sex of the baby**						
Female	1.00	–	1.00	–	1.00	–
Male	0.99	0.69, 1.43	0.57	0.39, 0.83	2.01	1.58, 2.54
**Antenatal depressive symptoms (EDS score)**						
< 10	1.00	–	1.00	–	1.00	–
≥ 10	1.22	0.79, 1.89	1.39	0.91, 2.15	1.12	0.84, 1.49

The unadjusted associations of baseline characteristics with the outcomes of interest are shown in Table 2. The ORs obtained from the logistic regression models indicated that women with antenatal depressive symptoms had higher odds of PTB, SGA, and LGA though none of these associations reached statistical significance. When we controlled for possible confounders, these associations became slightly stronger for PTB and LGA but they remained non-significant (Table 3). None of the factors examined (nationality and pre-pregnancy BMI) was found to be a modifier of the effect of antenatal depressive symptoms on the outcomes of interest. In the adjusted analysis, the risk of PTB was associated with conception by IVF (OR = 3.32, 95% CI: 1.18, 9.39) and preterm delivery in previous pregnancies (OR = 3.92, 95% CI: 1.99, 7.73) whereas the risk of SGA was associated with the sex of the baby, with males having lower odds of being small for gestation age than females (OR = 0.57, 95% CI: 0.39, 0.84). On the other hand, a male baby was a significant predictor of LGA (OR = 2.07, 95% CI: 1.56, 2.74). Non-Kuwaiti women had increased odds of delivering a LGA baby (OR = 1.92, 95% CI: 1.19, 3.08).

**Table 3:** Multivariable regression models for the outcomes of interest- Adjusted ORs

	aOR	95% CI	***p-***value
***PTB***^a^			
**In vitro fertilization**			0.02
No	1.00	–	
Yes	3.32	1.18, 9.39	
**Preterm delivery in previous pregnancies**			< 0.01
No previous pregnancy	1.00	–	
No preterm delivery	0.78	0.43, 1.42	
At least one preterm delivery	3.92	1.99, 7.73	
**Antenatal depressive symptoms (EDS score)**			0.22
< 10	1.00	–	
≥ 10	1.41	0.81, 2.45	
***SGA***^†^			
**Sex of the baby**			< 0.01
Female	1.00	–	
Male	0.57	0.39, 0.84	
**Antenatal depressive symptoms (EDS score)**			0.32
< 10	1.00	–	
≥ 10	1.26	0.80, 1.98	
***LGA***^b^			
**Nationality**			0.01
Kuwaiti	1.00	–	
Non- Kuwaiti	1.92	1.19, 3.08	
**Sex of the baby**			< 0.01
Female	1.00		
Male	2.07	1.56, 2.74	
**Antenatal depressive symptoms (EDS score)**			0.18
< 10	1.00	–	
≥ 10	1.27	0.90, 1.79	

^a^Further adjusted for nationality, household income, pre-pregnancy BMI group, gestational hypertension, and gestational diabetes

^†^Further adjusted for household income, pre-pregnancy BMI group, and education

^b^Further adjusted for household income, pre-pregnancy BMI group, gestational hypertension, gestational diabetes, age group, employment status, parity, and preterm delivery in previous pregnancies

Furthermore, we examined the strength of the linear association of antenatal depressive symptoms with birth weight and gestational length as continuous variables. The correlation between the EDS score and gestational length was r_s_ = − 0.02 (*p* = 0.39) and that of EDS and birth weight was r_s_ = − 0.04 (*p* = 0.07).

## Discussion

This study examined the association of depressive symptoms during pregnancy and adverse birth outcomes in a sample of pregnant women in Kuwait who were enrolled in the TRACER cohort. Overall, higher rates of preterm birth, as well as for small and large for gestational age babies, were found among women with an EDS score ≥ 10. However, none of these associations reached statistical significance either in univariate models or in multivariable models after controlling for socio-demographic and pregnancy related variables. These results are in line with studies conducted elsewhere, which showed that depression is not predictive of PTB, especially in women who are at a lower obstetric risk [34, 35]. On the other hand, in a recently published meta-analysis the pooled estimate from 14 studies with a total sample of 21,048 women showed that there is a much stronger association between clinical depression that requires treatment and adverse outcomes [9]. However, as Bindt et al. suggested, it seems that studies that had taken into consideration pregnancy complications did not find a strong association between depression and PTB, while many published studies which had shown a significant association failed to include these complications as risk factors of PTB [35]. In our analysis, we tried to include complications in current or previous pregnancies that may have increased the risk of adverse birth outcomes, to the extent that data were available.

A larger body of evidence exists on the effect of depression on low birth weight; in contrast, not many studies have examined the effect of depression on birth weight in relation to gestational age at delivery. We demonstrated that the levels of SGA were not different among women with and without depressive symptoms, which was consistent with some studies but inconsistent with others [15, 36]. Molyneaux et al. reported a weak impact of depression on SGA in obese and overweight women, though not in normal weight women. However, in our study, BMI was not shown to be an effect-modifier of this association [37].

The evidence regarding the effect of depression on delivering a large for gestational age baby is scarce. This lack is also noted in Jarde et al. [9]. Despite the fact that LGA was relatively common in our study, with more than a fifth of women delivering a LGA baby, we did not find any significant association between this outcome and depressive symptoms in pregnancy, in-line with the limited studies identified in the literature [38, 39]. Two other studies, which investigated the combined effect of a BMI 25.0 kg/m^2^ or higher and depression, reported a higher frequency of LGA babies or babies with greater standardized birthweight among women with both comorbidities [40, 41]. Similar to SGA, our results did not suggest that BMI modifies the association between depressive symptoms and LGA. Delivering a baby that has a weight that is greater for its gestational age is usually not considered as an adverse birth outcome but evidence suggests that it can be as unhealthy as SGA. Several studies have suggested that LGA, or macrosomia, can have a sequelae of adverse events at birth, childhood or adolescence [4, 7, 42]. Therefore, more research is needed to further examine the association of antenatal depression and large for gestational age.

We recognize some limitations in the analyses. The EDS was not validated in Kuwait, as this was not the main exposure of interest when setting up the cohort. An Arabic version has been validated in other Arab countries, however, the cultural setting of these countries could be different than Kuwait, which ranks among the richest countries in the world. Gestational age at birth was computed as the number of weeks since the last reported menstrual period and was not ascertained using ultrasound measurements. However, this is the most common method in practice to calculate gestational age in the absence of early pregnancy ultrasound data. Birth weight and birth dates were self-reported, which could potentially be subject to recall bias, though it is unlikely that any recall bias exists given the fact that mothers recall birth dates and weight very accurately. In addition, information was collected by a phone interview, therefore women had probably access to the health card of their baby. Finally, as there was limited information about the mode of delivery we could not adjust for it in the multivariable models.

Despite these limitations, the study has several strengths. First, the analysis is based on a prospective cohort study with a large sample size and a wide range of covariates considered. Furthermore, the data have been obtained from a population that has not been studied in the past, as regards to the role of mental health and adverse perinatal outcomes. Finally, the women recruited in the study included both Kuwaitis and non-Kuwaitis, who attended public and private clinics in all six governorates of Kuwait, in a way that represented the heterogeneous composition of the population of Kuwait. Therefore, the findings could be generalizable to the population of Kuwait and possibly to other countries that share similar biological and cultural characteristics.

## Conclusions

In conclusion, our study suggests that depressive symptoms in pregnancy did not predict adverse birth outcomes, such as PTB and SGA, which adds to the currently non-conclusive literature. Similarly, our findings showed that antenatal depressive symptoms are not associated with LGA though further research is needed to examine this association, given that the available evidence is quite limited.

## Data Availability

Permission to use data was obtained from the principal investigator. Restrictions apply regarding the availability of these data, which were used under license for the current study and thus are not publicly available. Data could be available upon reasonable request, after obtaining permission from the Dasman Diabetes Institute.

## Abbreviations

EDS: Edinburgh Depression Scale
EPDS: Edinburgh Postnatal Depression Scale
IVF: In-vitro fertilization
KWD: Kuwaiti Dinar
LBW: Low birth weight
LGA: Large for gestational age
PTB: Preterm birth
PTSD: Post-traumatic stress disorder
SGA: Small for gestational age
TRACER: Transgenerational Assessment of Children’s Environmental Risk
USD: United States Dollar

## References

[1] Blencowe H, Cousens S, Oestergaard MZ, Chou D, Moller A-B, Narwal R (2012). National, regional, and worldwide estimates of preterm birth rates in the year 2010 with time trends since 1990 for selected countries: a systematic analysis and implications. Lancet..

[2] Blencowe H, Cousens S, Chou D, Oestergaard M, Say L, Moller A-B (2013). Born too soon: the global epidemiology of 15 million preterm births. Reprod Health.

[3] Jelliffe-Pawlowski L, Baer R, Blumenfeld Y, Ryckman K, O’Brodovich H, Gould J (2015). Maternal characteristics and mid-pregnancy serum biomarkers as risk factors for subtypes of preterm birth. BJOG An Int J Obstet Gynaecol.

[4] Grissom NM, Reyes TM (2013). International journal of developmental neuroscience gestational overgrowth and undergrowth affect neurodevelopment : similarities and differences from behavior to epigenetics. Int J Dev Neurosci.

[5] Longo S, Bollani L, Decembrino L, Di Comite A, Angelini M, Stronati M (2013). Short-term and long-term sequelae in intrauterine growth retardation (IUGR). J Matern Neonatal Med.

[6] Saigal S, Doyle LW (2008). An overview of mortality and sequelae of preterm birth from infancy to adulthood. Lancet..

[7] Chiavaroli V, Marcovecchio ML, de Giorgis T, Diesse L, Chiarelli F, Mohn A (2014). Progression of cardio-metabolic risk factors in subjects born small and large for gestational age. PLoS One.

[8] Grote N, Bridge J, Gavin A, Melville J, Iyengar S, Katon W (2010). A meta-analysis of depression during pregnancy and the risk of preterm birth, low birth weight, and intrauterine growth restriction. Arch Gen Psychiatry.

[9] Jarde A, Morais M, Kingston D, Giallo R, Macqueen GM, Giglia L (2016). Neonatal outcomes in women with untreated antenatal depression compared with women without depression a systematic review and meta-analysis. JAMA Psychiatry.

[10] Grigoriadis S, VonderPorten EH, Mamisashvili L, Tomlinson G, Dennis CL, Koren G (2013). The impact of maternal depression during pregnancy on perinatal outcomes: a systematic review and meta-analysis. J Clin Psychiatry.

[11] Fransson E, Örtenstrand A, Hjelmstedt A (2011). Antenatal depressive symptoms and preterm birth: a prospective study of a Swedish national sample. Birth..

[12] Li D, Liu L, Odouli R (2009). Presence of depressive symptoms during early pregnancy and the risk of preterm delivery : a prospective cohort. Hum Reprod.

[13] Niemi M, Falkenberg T, Petzold M, Chuc NTK, Patel V (2013). Symptoms of antenatal common mental disorders, preterm birth and low birthweight: a prospective cohort study in a semi-rural district of Vietnam. Tropical Med Int Health.

[14] Straub H, Adams M, Kim JJ, Silver RK (2012). Antenatal depressive symptoms increase the likelihood of preterm birth. Am J Obstet Gynecol.

[15] Szegda K, Bertone-Johnson ER, Pekow P, Powers S, Markenson G, Dole N (2016). Depression during pregnancy and adverse birth outcomes among predominantly Puerto Rican women. Matern Child Health J.

[16] Berle JØ, Mykletun A, Daltveit AK, Rasmussen S, Holsten F, Dahl AA (2005). Neonatal outcomes in offspring of women with anxiety and depression during pregnancy. A linkage study from the Nord-Trøndelag health study (HUNT) and medical birth registry of Norway. Arch Womens Ment Health.

[17] Eastwood J, Ogbo FA, Hendry A, Noble J, Page A (2017). The impact of antenatal depression on perinatal outcomes in Australian women. PLoS One.

[18] Husain N, Munshi T, Jafri F, Husain M, Parveen A, Saeed Q (2014). Antenatal depression is not associated with low-birth weight: a study from urban Pakistan. Front Psychiatry.

[19] Varela P, Spyropoulou AC, Kalogerakis Z, Moraitou M, Zervas IM. Limited depressive and anxiety symptoms late in pregnancy are not related to neonatal outcomes. Nurs Midwifery Stud. 2015;4. 10.17795/nmsjournal29308. 10.17795/nmsjournal29308 PMC4644606 26576444

[20] Neggers Y, Goldenberg R, Cliver S, Hauth J (2006). The relationship between psychosocial profile, health practices, and pregnancy outcomes. Acta Obstet Gynecol Scand.

[21] Bolten MI, Wurmser H, Buske-Kirschbaum A, Papoušek M, Pirke KM, Hellhammer D (2011). Cortisol levels in pregnancy as a psychobiological predictor for birth weight. Arch Womens Ment Health.

[22] Goldenberg RL, Culhane JF, Iams JD, Romero R (2008). Epidemiology and causes of preterm birth. Lancet..

[23] Zuckerman B, Amaro H, Bauchner H, Cabral H (1989). Depressive symptoms during pregnancy: relationship to poor health behaviors. Am J Obstet Gynecol.

[24] Yonkers KA, Smith MV, Forray A, Epperson CN, Costello D, Lin H (2014). Pregnant women with posttraumatic stress disorder and risk of preterm birth. JAMA Psychiatry.

[25] Shapiro GD, Fraser WD, Frasch MG, Séguin JR (2013). Psychosocial stress in pregnancy and preterm birth: associations and mechanisms. J Perinat Med.

[26] AlSeaidan M, Al Wotayan R, Christophi CA, Al-Makhseed M, Abu Awad Y, Nassan F (2016). Birth outcomes in a prospective pregnancy-birth cohort study of environmental risk factors in Kuwait: the TRACER study. Paediatr Perinat Epidemiol.

[27] Pampaka D, Papatheodorou SI, AlSeaidan M, Al Wotayan R, Wright RJ, Buring JE (2018). Depressive symptoms and comorbid problems in pregnancy - results from a population based study. J Psychosom Res.

[28] World Health Organization (1977). WHO: recommended definitions, terminology and format for statistical tables related to perinatal period and use of a new certificate for cause of perinatal deaths. Acta Obstet Gynecol Scand.

[29] World Health Organization. Weight percentiles calculator. https://www.who.int/reproductivehealth/topics/best_practices/weight_percentiles_calculator.xls. Accessed 23 Oct 2020.

[30] Battaglia FC, Lubchenco LO (1967). A practical classification of newborn infants by weight and gestational age. J Pediatr.

[31] Matsumura K, Hamazaki K, Tsuchida A, Kasamatsu H, Inadera H, Kamijima M (2020). Factor structure of the Edinburgh postnatal depression scale in the Japan environment and Children’s study. Sci Rep.

[32] Nasreen HE, Kabir ZN, Forsell Y, Edhborg M (2010). Low birth weight in offspring of women with depressive and anxiety symptoms during pregnancy: results from a population based study in Bangladesh. BMC Public Health.

[33] Shakeel N, Eberhard-Gran M, Sletner L, Slinning K, Martinsen EW, Holme I (2015). A prospective cohort study of depression in pregnancy, prevalence and risk factors in a multi-ethnic population. BMC Pregnancy Childbirth.

[34] Gawlik S, Waldeier L, Müller M, Szabo A, Sohn C, Reck C (2013). Subclinical depressive symptoms during pregnancy and birth outcome--a pilot study in a healthy German sample. Arch Womens Ment Health.

[35] Bindt C, Guo N, Te Bonle M, Appiah-Poku J, Hinz R, Barthel D (2013). No association between antenatal common mental disorders in low-obstetric risk women and adverse birth outcomes in their offspring: results from the CDS study in Ghana and Côte D’Ivoire. PLoS One.

[36] Smith KF, Brunner LR, Michele HL, Warren-findlow J. The association between maternal depression during pregnancy and adverse birth outcomes : a retrospective cohort study of PRAMS participants. J Community Health. 2015:984–92. 10.1007/s10900-015-0022-4. 10.1007/s10900-015-0022-4 25833420

[37] Molyneaux E, Pasupathy D, Poston L, Howard LM, On behalf of the SCOPE Consortium (2015). Antenatal depression, preeclampsia and small for gestational age delivery (SGA) in overweight and obese pregnant women. Psychoneuroendocrinology.

[38] Pesonen AK, Lahti M, Kuusinen T, Tuovinen S, Villa P, Hämäläinen E (2016). Maternal prenatal positive affect, depressive and anxiety symptoms and birth outcomes: the PREDO study. PLoS One.

[39] Li X, Gao R, Dai X, Liu H, Zhang J, Liu X (2020). The association between symptoms of depression during pregnancy and low birth weight: a prospective study. BMC Pregnancy Childbirth.

[40] Mcdonald SD, Mckinney B, Foster G, Taylor V, Lutsiv O, Pullenayegum E (2015). The combined effects of maternal depression and excess weight on neonatal outcomes.

[41] Petursdottir H, Skalkidou A, Sjöholm A, Eurenius-orre K, Mulic-lutvica A, Wikström A (2019). Maternal body mass index moderates antenatal depression effects on infant birthweight.

[42] Fuchs F, Bouyer J, Rozenberg P, Senat M-V (2013). Adverse maternal outcomes associated with fetal macrosomia: what are the risk factors beyond birthweight?. BMC Pregnancy Childbirth.

